# Linking Global Integrated Assessment and Hybrid Input–Output Models To Support Steel Decarbonization Policies in Europe

**DOI:** 10.1021/acs.est.5c15099

**Published:** 2026-06-09

**Authors:** Debora Ghezzi, Lorenzo Rinaldi, Russell Horowitz, Dirk-Jan van de Ven, Georg Holtz, Alexander Jülich, Matteo Vincenzo Rocco

**Affiliations:** † Department of Energy, 18981 Politecnico di Milano, 20156, Milan, Italy; ‡ 202620 Basque Center for Climate Change, 48940, Leoia, Spain; § Wuppertal Institute for Climate, Environment & Energy, 42103, Wuppertal, Germany

**Keywords:** steel decarbonization, carbon footprint, input−output analysis, integrated assessment modeling, CBAM, GCAM, Exiobase

## Abstract

Formulating decarbonization strategies for the European steel industry is crucial for meeting EU climate goals while preserving competitiveness. The present study develops an integrated modeling framework linking GCAM, a technology-rich global integrated assessment model, with the MARIO multiregional input–output framework to trace EU steel production, trade, and its direct and embedded greenhouse-gas emissions. Four scenarios are examined: a baseline aligned with current pledges and three pathways resembling major EU policy packages for 2025–2050, notably the Carbon Border Adjustment Mechanism (CBAM) and green-steel subsidy schemes. Results indicate that CBAM promotes low-emissions steel technologies, reduces total embedded emissions in EU steel consumption (up to −46% in 2030 and −23% in 2050), and helps stabilize the sector during the transition. Complementary green-steel support accelerates deployment of low-carbon production capacity and strengthens long-run competitiveness. Across scenarios, emission intensities of steel production fall (up to −80% in 2050), and consumption-based footprints associated with EU steel demand decline. The GCAM–MARIO coupling provides a transparent link between scenario design and economy-wide footprint accounting, illustrating how border measures and targeted subsidies interact. Overall, combining CBAM with sustained support for green-steel technologies offers an effective pathway to decarbonize the European steel industry while maintaining its international competitiveness.

## Graphical abstract

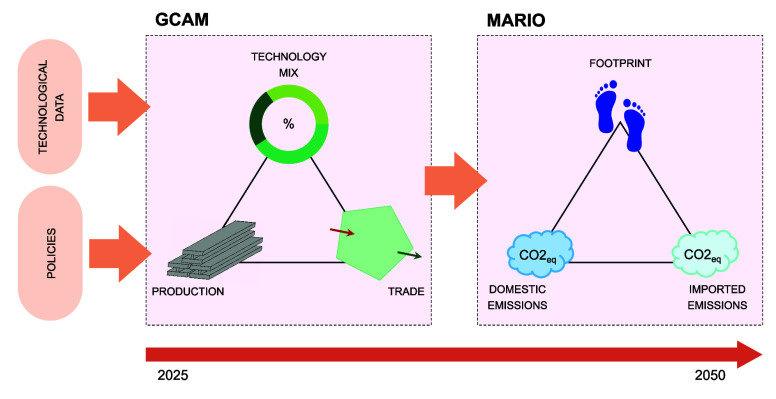

## 1 Introduction

The decarbonization of the European steel industry is central to the EU’s long-term climate ambitions and industrial competitiveness.^1^ Steel production currently accounts for approximately 5% of the European Union’s greenhouse gas (GHG) emissions,^2^ largely owing to the predominance of the emissions-intensive blast furnace/basic oxygen furnace (BF-BOF) route.^3^ Transitioning to low-carbon steel technologies, notably hydrogen-based Direct Reduction (DR) and enhanced scrap recycling via Electric Arc Furnaces (EAF), has therefore emerged as a strategic priority in EU industrial and climate policy. Recent policy developments, including the tightening of the EU Emissions Trading System (ETS) and the phasing out of free CO_2_ allowances, encourage the European steel industry to accelerate this transition. However, these measures raise acute concerns about carbon leakage and production relocation: as decarbonization costs rise, primary steel production may shift to regions with less stringent climate policies, potentially resulting in higher global GHG emissions.

To address the tension between relocation risk and ambitious climate targets, the EU developed the Carbon Border Adjustment Mechanism (CBAM).^4^ Unlike conventional carbon pricing, which operates within domestic markets, CBAM represents the first large-scale attempt to deploy trade regulation for two distinct purposes: preserving fair competition among industries subject to unequal carbon pricing obligations and sending an indirect signal to EU trade partners to improve their environmental performance. By conditioning market access on the embodied carbon content of imported steel, the mechanism aims to reduce carbon leakage while simultaneously encouraging the consumption of low-carbon steel within the EU. While CBAM prioritizes reducing high-emission steel imports, European steel industries are advocating for additional, more targeted measures to support domestic low-carbon steel production. These include incentives for capital investment and operational cost reduction.^5^

A growing body of modeling studies, predominantly drawing on economic frameworks, has emerged in the literature to examine the potential impacts of CBAM not only on the EU steel sector, but also on the steel industries of non-EU exporting countries. This broader scope reflects the far-reaching influence of EU trade policy: as the world’s largest steel importer,^6^ the EU represents a critical market whose regulatory decisions inevitably reverberate across the global steel industry. For instance, Shuai et al.^7^ investigated the global effects of CBAM on GDP and CO_2_ emissions in the steel sector using an input–output-based Computable General Equilibrium (CGE) model, while Bellora and Fontagnè^8^ employed a dynamic CGE model capturing input–output relations and GHG emissions to assess CBAM’s potential economic effects and its capacity to reduce carbon leakage. Li et al.^9^ took a different approach, combining input–output models with machine learning to estimate reductions in embodied carbon in global steel trade under CBAM scenarios without free allowances.

Beyond global-scope analyses, a parallel strand of research has examined CBAM’s impacts on specific countries or country groups. For instance, Shin et al.^10^ compared emission estimates derived from the GTAP and OECD ICIO databases to inform the design of more equitable CBAM tariffs for South Korea. Magacho et al.,^11^ employing a multiregional input–output model, demonstrated that CBAM could significantly impair the economic performance of many developing countries. Das and Bandyopadhyay^12^ adopted a qualitative methodology to assess CBAM’s potential role in promoting decarbonization in the Indian steel industry. The largest share of country-specific studies, however, focuses on China, the world’s leading steel exporter.^6^ For instance, Wang et al.^13^ utilized the GTAP-E model to analyze CBAM’s effect on the competitiveness of Chinese export trade, with particular attention to carbon-intensive industries such as iron and steel, while Zhao et al.^14^ assessed the impact of CBAM on China’s steel exports and carbon emissions through a system dynamics model.

The majority of these studies rely on macroeconomic models mainly based on input–output databases, that lack technological granularity, often representing only a limited set of steelmaking routes. Horowitz et al.^15^ addressed this limitation by expanding the steel sector representation within the GCAM model to encompass a broader range of production technologies, analyzing the impact of CBAM on steel production, consumption, and price across OECD and non-OECD countries. Despite its technological richness, however, this study does not link production and consumption volumes with a comprehensive accounting of the GHG emissions embedded in the EU steel consumption under CBAM, which is essential for evidence-based policymaking given that one of the CBAM’s core objectives is to reduce the GHG footprint of steel consumed within the EU. To the authors’ knowledge, the only study combining technological detail with comprehensive supply chain tracing is Pan et al.,^16^ who coupled the GCAM-China integrated assessment model with a global multiregional input–output table to project the export cost implications of CBAM for Chinese provinces. Their analysis, however, remains confined to the economic implications and focuses on China, leaving a substantial gap in the literature for integrated approaches that jointly assess the EU steel sector’s environmental and trade performance under CBAM.

The present study aims therefore to quantify the effects of CBAM on the EU steel sector in two complementary ways: first, by analyzing the trade balance to determine how CBAM influences EU imports and supports domestic production; and second, by calculating the climate footprint to assess whether the policy effectively steers production toward lower-emission steel. The study further investigates the potential effects of combining CBAM with additional incentive mechanisms for green steel production technologies on the EU domestic output.

To achieve these objectives, the study adopts an integrated modeling approach, coupling the global scenario capabilities of the GCAM Integrated Assessment Model^17^ with the MARIO input–output framework.^18^ This coupling enables the consistent linking of technology-explicit transition pathways with supply chain emission accounting, extending the work of Rinaldi et al.,^19^ who assessed the environmental impacts of exogenously defined EU steel sector decarbonization pathways using a technology-rich MRIO model.

The following sections are organized as follows: Section 2 describes the modeling framework adopted for the analysis, detailing the GCAM-MARIO integration and presenting the scenarios examined; section 3 presents results on steel production, consumption, and trade, along with their associated environmental impacts, and discusses the findings.

## 2 Materials and Methods

### 2.1 Models-Coupling Workflow

The models coupling developed for the present study consists of the soft link of GCAM,^20^ a multisector global integrated assessment model, and a multiregional Leontief input-output model^21^ based on the environmentally extended Exiobase hybrid-units database.^22^ GCAM provides a technology-rich representation of the economy, energy system, agriculture, and water supply across 32 geopolitical regions, while Exiobase encompasses 200 products and 163 industries across 44 individual countries and 5 rest-of-the-world regions.

Drawing on detailed technological data and subject to the constraints imposed by the policy scenarios under investigation, GCAM determines the cost-optimal combination of steel production, consumption, exports, and imports in each region, thereby identifying regional decarbonization pathways up to 2050. Furthermore, GCAM specifies the regional power and steel supply mixes for each modeling year. These mixes, describing how steel and power production evolve in each region over time, are subsequently incorporated into the Exiobase database. The input–output table itself remains structurally unchanged; only the steel and power production mixes are updated in line with the endogenous results provided by GCAM. Using the Exiobase database incorporating the updated mixes, year- and technology-specific carbon footprints of steel are then computed, alongside the total emissions embedded in steel consumption. These calculations are performed specifically for the European Union, which is the region of primary interest in this study. All interactions with the Exiobase database (e.g., incorporation of new production mixes, computation of footprints) are carried out using MARIO,^23^ an open-source Python package for the manipulation of input-output tables and models.

The soft-linking procedure between the two models is illustrated schematically in Figure 1. The mathematical formulations underlying GCAM and MARIO are detailed in external references,^17,23^ while additional methodological details and the complete set of extended results are provided in the Supporting Information.

**Figure 1:**
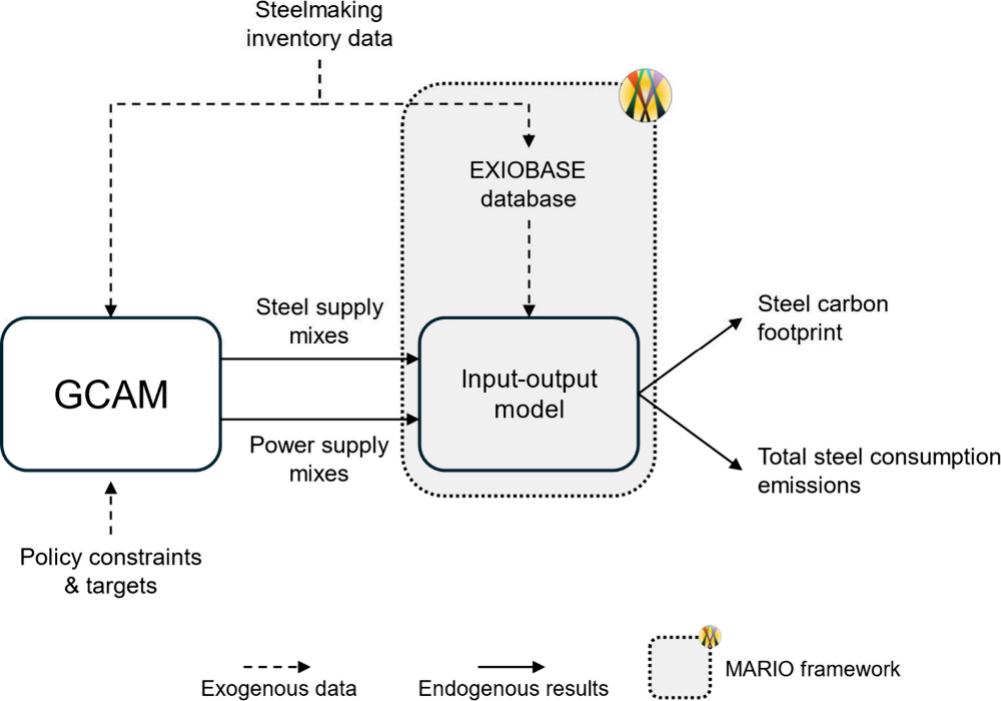
Modeling framework workflow.

### 2.2 Integration of Additional Steelmaking Technologies in GCAM and Exiobase

The GCAM model and the MARIO framework rely on intrinsically different methodologies; it is therefore necessary to ensure consistent assumptions about the input data and to define an appropriate mapping of technological and regional scopes for the variables involved in the linking process.

Regarding steelmaking technologies, the nine production routes originally included in GCAM 7.0 are expanded, following the methodology outlined in Horowitz et al.,^15^ to encompass the routes characterized in the technology inventory of Zelt et al.^24^ While the underlying technology inventory focuses on crude steel production, the present study extends the system boundaries to include the steel finishing stage. This extension allows for the consideration of internal utilization of process off-gases in hot rolling. Finishing input demands have been adapted from UBA.^25^ Detailed assumptions regarding the finishing energy demands and the corresponding data derivations are provided in the Supporting Information. The inventory in Zelt et al. is further expanded to include additional technologies, derived from Bilici et al.^26^ and Agora Industry^27^ through specific parameter variations. With these modifications, the final inventory adopted in this study comprises 22 steelmaking routes. As for MARIO, the raw Exiobase database^28^ originally distinguishes only two steelmaking routes: one for primary steel, mostly reflecting the BF-BOF process, and one for secondary steel via scrap-based EAF. To ensure a consistent technological characterization across both models, Exiobase is expanded to include all additional routes considered in GCAM, following the methodological approach outlined in Rinaldi et al.^19^ and drawing on the same technology inventory used to extend GCAM. One asymmetry remains, however: while GCAM distinguishes four separate secondary steel production technologies, MARIO adopts a simplified representation by aggregating these into a single secondary route. The full set of steelmaking routes included in the analysis, together with their direct emissions, is reported in Table 1 (the “Emissions category” column is discussed in section 2.3.1).

**Table 1:** Direct Emissions and Emissions Category of All Steelmaking Routes Analyzed in the Study^a^

technology	acronym	direct emissions [kgCO_2_/ton]	emissions category
Blast Furnace-Basic Oxygen Furnace	BF-BOF	2183	High
Blast Furnace-Basic Oxygen Furnace + CCS 73%	BF-BOF-CCS-73%	590	Medium
Blast Furnace-Basic Oxygen Furnace + CCS 86%	BF-BOF-CCS-86%	306	Low
Blast Furnace-Basic Oxygen Furnace with bioenergy (min) + CCS	BF-BOF-BECCSmin	<0	Low
Blast Furnace-Basic Oxygen Furnace with bioenergy (max) + CCS	BF-BOF-BECCSmax	<0	Low
Smelting Reduction-Basic Oxygen Furnace	SR-BOF	1476	High
Smelting Reduction-Basic Oxygen Furnace + CCS	SR-BOF-CCS	207	Low
Direct Reduction of Iron with Natural Gas-Electric Arc Furnace	DRI-EAF-NG	736	Medium
Direct Reduction of Iron with Natural Gas-Electric Arc Furnace + CCS	DRI-EAF-NG-CCS	199	Low
Direct Reduction of Iron with Coal-Electric Arc Furnace	DRI-EAF-COAL	2286	High
Direct Reduction of Iron with Coal-Electric Arc Furnace + CCS	DRI-EAF-COAL-CCS	320	Low
Direct Reduction of Iron with Bioenergy-Electric Arc Furnace + CCS	DRI-EAF-BECCS	<0	Low
Direct Reduction of Iron with (Green) Hydrogen-Electric Arc Furnace	DRI-EAF-H2	0	Low
Direct Reduction of Iron with Natural Gas-Submerged Arc Furnace-Basic Oxygen Furnace	DRI-SAF-BOF-NG	704	Medium
Direct Reduction of Iron with Hydrogen-Submerged Arc Furnace-Basic Oxygen Furnace	DRI-SAF-BOF-H2	0	Low
Direct Reduction of Iron with Bioenergy-Submerged Arc Furnace-Basic Oxygen Furnace + CCS	DRI-SAF-BOF-BECCS	<0	Low
Alkaline Electrolysis-Electric Arc Furnace	AEL-EAF	0	Low
Molten Oxide Electrolysis	MOE	0	Low
Electric Arc Furnace (fossil) and finishing with Natural Gas*	EAF-fossil-NG*	199	Low
Electric Arc Furnace (bioenergy) and finishing with Natural Gas*	EAF-bio-NG*	136	Low
Electric Arc Furnace (fossil) and electrical finishing*	EAF-fossil-EL*	64	Low
Electric Arc Furnace (bioenergy) and electrical finishing*	EAF-bio-EL*	0	Low

a Values expressed per tonne of finished steel. Technologies marked with * denote secondary steel production routes.

A key challenge in the linking process concerns the differing levels of regional aggregation for the European Union: GCAM divides the EU into two clusters (EU-12 and EU-15), whereas the Exiobase database distinguishes all 27 member states individually. To reconcile this discrepancy, the European regions in Exiobase are aggregated employing MARIO to align with GCAM’s regional structure. The regional scopes of both models and the concordance tables used for their alignment are provided in the Supporting Information.

The analysis covers the period 2025–2050 in five-year time steps. GCAM is hard-calibrated with historical data up to 2015, while relevant outputs for the European steel industry (e.g., production by route, imports, and emissions) are soft-calibrated for 2020.

### 2.3 Scenarios Formulation

The GCAM-MARIO integrated modeling framework is applied to four different scenarios reflecting varying degrees of policy ambition to balance competitiveness, emissions reduction, and industrial transformation within the global steel sector:

1. **NDC_LTT**.
  This scenario, serving as the baseline, assumes unrestricted global trade with all countries participating in the global market. Each country sets its own CO_2_ price to meet the climate targets outlined in its Nationally Determined Contributions (NDCs) and long-term targets (LTT), while market forces determine the location and technology of steel production. Regional differences in carbon pricing affect the competitiveness of the steel sector, driving shifts in global production patterns. Specifically, EU steel emissions are priced at 30% of the EU Emissions Trading System (ETS) value in 2020, rising to 45% in 2025 and 60% by 2030, with remaining emissions covered by free allowances. Non-EU regions face no steel-related carbon costs until 2035.
2. **CBAM**.
  This scenario extends the baseline by introducing CBAM in the EU from 2030 onward. This mechanism is initially priced at 60% of the ETS value and reaches full ETS parity (100%) by 2035. All non-EU countries continue to behave as in the NDC_LTT scenario with open trade maintained.
3. **CBAM-G_SUBS**.
  While CBAM incentivizes the consumption of low-emission steel, it does not guarantee that such steel is produced domestically in the EU. To support the competitiveness of the European industry as it transitions to clean production, the steel sector has called upon the EU to allocate financial resources directly toward decarbonization projects.^29^ This scenario therefore combines CBAM with EU-level capital cost subsidies for low-carbon primary steelmaking technologies. Subsidies are set at 30% in 2030 and reduced to 10% by 2040. This type of support is aimed at reducing upfront investment costs and addressing financial barriers associated with the deployment of capital-intensive low-carbon technologies.
4. **CBAM-G_SUBS_HI**.
  This scenario follows the same logic as that of CBAM-G_SUBS but introduces a different form of financial support. Rather than capital subsidies, support is provided for nonenergy operational costs at a fixed rate of 20% over the period 2030–2050. By lowering operating rather than investment costs, these subsidies aim to enhance the cost competitiveness of low-carbon steel production.

In all scenarios, the deployment of scrap-based technologies is constrained to reflect estimated scrap availability and feasible usability of secondary steel (i.e., the amount of primary steel that can realistically be replaced by secondary steel due to quality requirements). Specifically, total global scrap-based steel production is limited in line with the scrap availability trajectory of the IEA Stated Policy Scenario (IEA STEPS).^30^ This assumption limits the share of secondary steelmaking to levels consistent with projected scrap generation, thereby precluding unrealistically high recycling rates in modeling years.

#### 2.3.1 International Trade and CBAM Modeling in GCAM

GCAM models international commodity trade using the Armington global trade approach.^31^ Rather than simulating explicit bilateral trade flows, this approach aggregates exports from all regions worldwide into a global trade pool. Each region can then source commodities either domestically or from the pooled international market. The adapted version of GCAM applied in this study disaggregates the single global trade pool for steel into six distinct pools, differentiated by direct emissions intensity (low, medium, high), as detailed in Table 1, and by origin (EU and non-EU). Historical exports and imports by emissions category are assumed to mirror production proportions, while the market split between EU and non-EU steel imports is calibrated using iron and steel trade data from Chatham House’s resourcetrade.earth platform.^32^

This trade modeling capability of GCAM is leveraged in this study to model CBAM. In principle, CBAM imposes a levy on imported goods, including steel, based on their embodied emissions, encompassing both direct emissions from production and indirect emissions from upstream energy and material inputs. Any carbon price paid in the region of origin is deducted from the CBAM cost imposed on each imported good.^33^ However, certain simplifications are required in CBAM modeling to maintain scientific consistency within the GCAM structure. While the CBAM regulation^4^ requires including not only suppliers’ direct emissions, but also their emissions related to electricity consumption and estimates from their own suppliers’ (i.e., their “precursors”), calculating these across complex international supply chains is practically challenging,^34,35^ especially for less-developed countries.^36–39^ Within the model, therefore, the levy applied to EU steel imports is based solely on the average direct emissions associated with each non-EU trade pool. Furthermore, since GCAM does not track specific bilateral trade flows, region-specific carbon prices paid by exporters cannot be individually deducted from import levies; instead, a global mean carbon adjustment is applied as an approximation (scenario- and year-specific values are reported in the Supporting Information).

Restricting the analysis to direct emissions and applying a unified carbon price adjustment allows the main policy levers to be evaluated within credible structural boundaries, while acknowledging that some degree of under- or overestimation may arise as global decarbonization and carbon pricing patterns evolve. Under current conditions, this approach is unlikely to introduce systematic bias in CBAM’s modeled effect:

- In early modeling years, when carbon pricing outside the EU remains limited and electricity-related emissions are relatively high, omitting indirect emissions may lead to a modest underestimation of CBAM costs for some trade pools.
- As decarbonization accelerates, the exclusion of indirect emissions becomes progressively less significant. However, the wider adoption of carbon pricing may cause the global average carbon adjustment to overestimate the CBAM levies.

Import allocations for linkage to the MARIO model follow the same logic: in the absence of bilateral trade flows, EU imports from individual regions are allocated in proportion to each region’s share of total exports to the appropriate trade pool. This approach enables MARIO to trace the evolving European steel supply chain in a manner that is fully transparent and consistent with the underlying GCAM trade structure.

## 3 Results and Discussion

The integration of GCAM and MARIO enables us to derive results on the quantities of steel produced, consumed, and traded within the EU, as well as their associated climate impacts. Production, consumption, and trade volumes are determined by GCAM for each analyzed scenario and are presented in section 3.1. The environmental impacts, by contrast, are derived using MARIO, which calculates the greenhouse gas (GHG) footprint both at the technological level (section 3.2.1) and at the scenario level (section 3.2.2), drawing on GCAM outputs for regional power and steel production mixes, as illustrated schematically in Figure 1.

### 3.1 GCAM Results: Steel Production, Consumption and Trades in the EU

The evolution of production, consumption, imports, and exports of steel across scenarios over the period 2025–2050 is illustrated in Figure 2.

**Figure 2:**
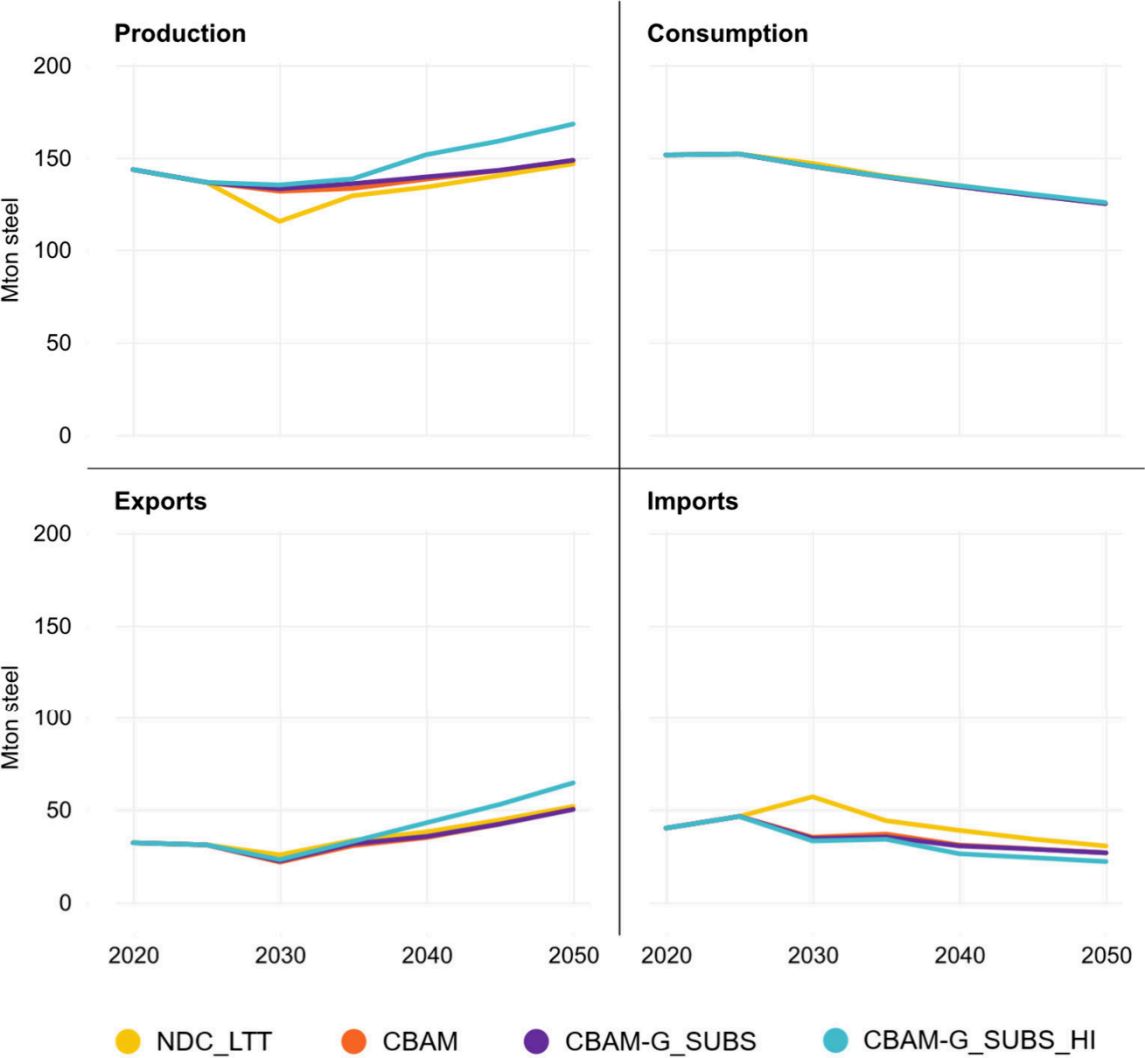
Steel consumption, production, and trade patterns in the EU in 2020–2050 by scenario.

In the NDC_LTT scenario, where no policy measures are designed to mitigate carbon leakage, the discrepancy in climate targets between the EU and other world regions in the short term is set to undermine the competitiveness of the European steel industry, as reflected in declining production and export levels and rising import penetration. EU steel production and exports decline by 19% and 20%, respectively, between 2020 and 2030. At the same time, EU consumption also declines, though not to a sufficient extent to offset the decline in production, resulting in a 42% increase in imports compared to 2020. Steel consumption, although endogenously determined by GCAM, is almost entirely driven by exogenous factors such as GDP and population growth, which are held consistent across all scenarios. Combined with the model’s low price-responsiveness, this explains why consumption levels remain nearly identical across scenarios. The introduction of CBAM moderates the contraction in production, which falls by only 6–8% between 2020 and 2030 across the three CBAM scenarios. Given similar consumption trajectories, this translates into a 12–17% reduction in imports over the same period. The near-identical short-term results across all three CBAM scenarios suggest that subsidies, whether on capital or operational costs, have no effect on EU steel cost competitiveness in the short term.

In the long term (2030–2050), the progressive introduction of effective CO_2_ pricing in other world regions is expected to raise global steel prices, thereby improving EU competitiveness even in the absence of additional policy instruments. Across all scenarios, EU steel production is projected to recover to approximately 2020 levels by 2050, notwithstanding a continued decline in domestic consumption. This recovery is underpinned by robust export growth, alongside relatively contained import levels. The most pronounced expansion in production and exports is observed in the CBAM-G_SUBS-HI scenario, where operational cost subsidies provide sustained support for green steel technologies. By contrast, the capital investment subsidies in the CBAM-G_SUBS scenario do not yield meaningfully higher production levels than those of CBAM alone.

Disaggregating by emissions intensity, production and exports of ‘high’ and ‘medium’ emissions steel are projected to decline sharply in the EU between 2020 and 2030 across all scenarios, driven by elevated CO_2_ costs. In the NDC_LTT scenario, this production decline is partially offset by a corresponding rise in imports of similarly emission-intensive steel. In the CBAM scenarios, by contrast, import levies substantially curtail imports of these categories, reducing their consumption levels within the EU accordingly.

Long-term projections point to a continued decline in ‘high’ and ‘medium’ steel production, accompanied by growth in ‘low’ emissions steel across both primary and secondary routes, as shown in Figure 3. ‘Medium’ steel production technologies see negligible adoption throughout the modeling horizon. This suggests that, when new steelmaking capacity is introduced, low-emission technologies are economically preferable to medium-emission alternative, as the latter remain subject to ETS costs owing to their insufficiently low emissions intensity. CBAM and capital cost subsidies accelerate the scale-up of low-carbon secondary steel production around 2030; however, this initial effect gradually dissipates, and production levels converge toward the baseline by the end of the modeling horizon. In the CBAM-G_SUBS_HI scenario, sustained operational cost subsidies support a more durable expansion of low-carbon primary steel production, driving higher export volumes from 2035 onward while domestic consumption remains stable.

**Figure 3:**
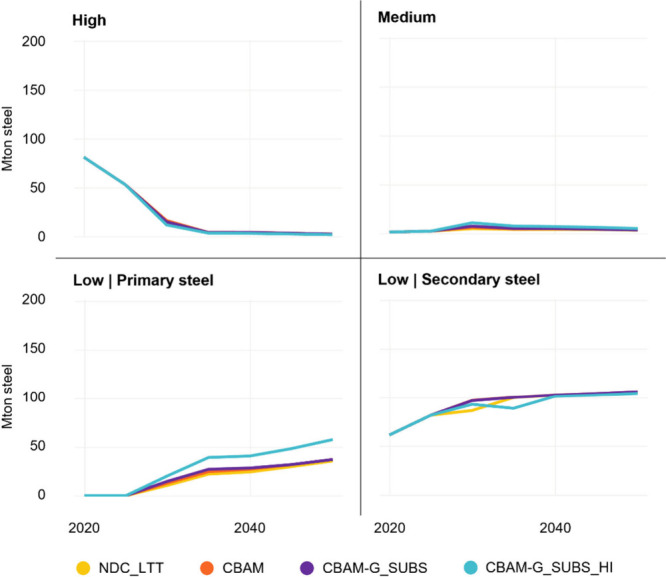
Steel production from high-, medium-, and low-emissions technologies in the EU by scenario. Low-emissions technologies are distinguished between primary and secondary steel production technologies.

Across all scenarios, the reduction in high-emission steel production is driven by the near-complete phase-out of the conventional BF-BOF route, whose share falls from 59% of total production in 2020 to just 0.3–0.5% in 2050. The only other high-emission technology deployed is SR-BOF, which accounts for at most 1.1% of total production in 2050. The third high-emission technology, DRI-EAF-COAL, is not deployed in any scenario across the entire modeling horizon and is notably the only one among the 22 technologies considered that remains unused. Medium-emission technologies contribute only marginally to the total output in all scenarios; among these, DRI-SAF-BOF-NG represents the most widely adopted configuration, albeit with a limited overall share.

Among low-emission technologies, scrap-based production routes form the backbone of decarbonized steel output across all scenarios. In the short term, all CBAM scenarios exhibit a higher penetration of alternative low-emission technologies, particularly DRI-EAF-based configurations, relative to the NDC_LTT scenario. Capital subsidies in the CBAM-G_SUBS scenario induce a moderate additional increase in DRI-EAF deployment compared with CBAM alone (6.19 Mton in 2030 versus 5.26 Mton); however, production levels converge in the long term, reaching approximately 10.40 Mton in 2050, a value comparable to that observed in the NDC_LTT scenario. By contrast, subsidies targeting nonenergy operational costs sustain a more durable expansion of DRI-EAF-based technologies, with output increasing from 9.49 Mton in 2030 to 16.98 Mton in 2050. More broadly, the CBAM-G_SUBS_HI scenario fosters a more diversified portfolio of low-emission steel technologies relative to the other scenarios, as illustrated in Figure 4.

**Figure 4:**
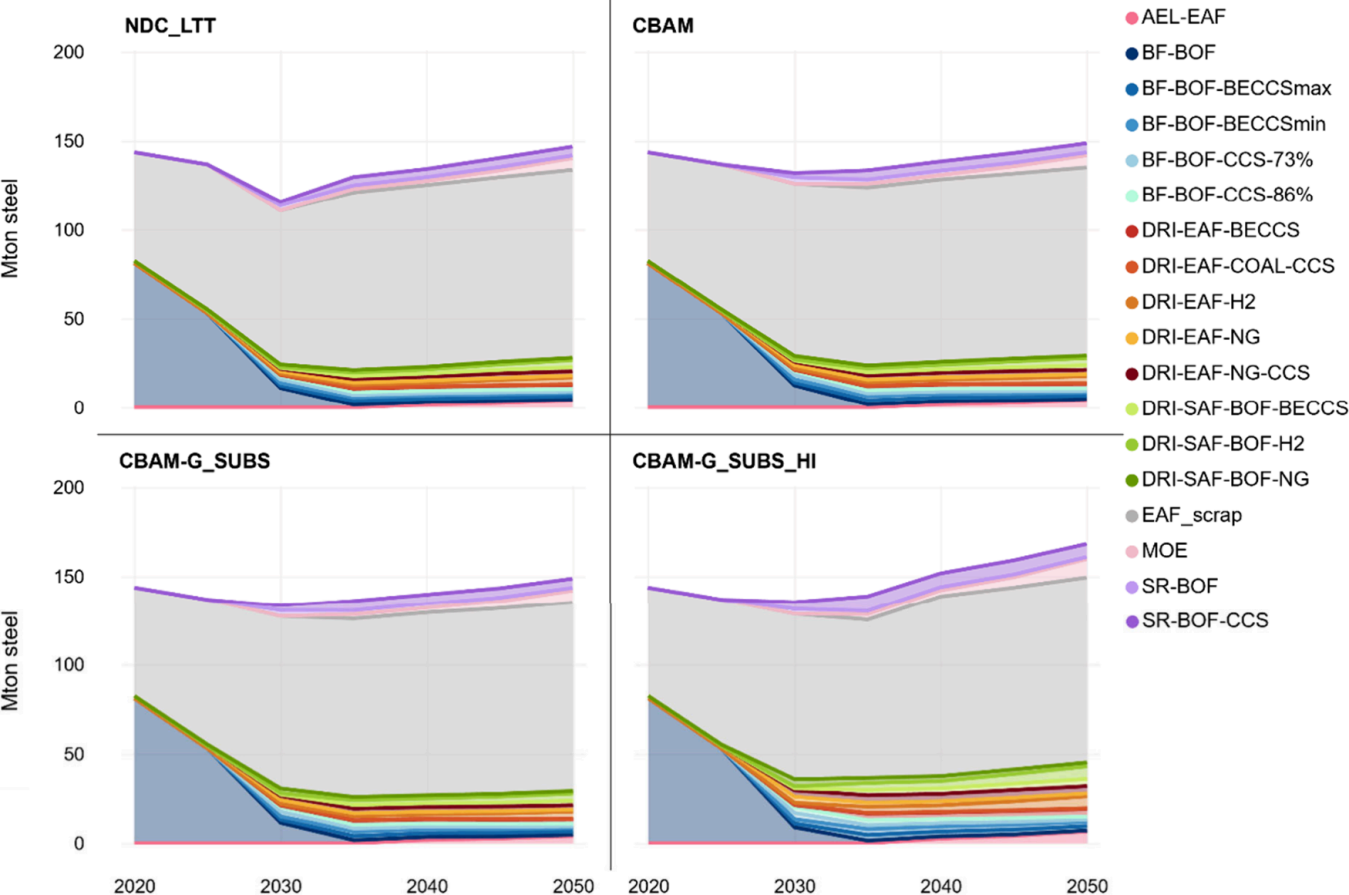
Steel production in the EU by technology, scenario and year.

### 3.2 MARIO Results: Environmental Impact Assessment

#### 3.2.1 Environmental Impact of the Steel Technologies

The GHG footprints of individual steel production routes in the EU, as calculated using MARIO, are illustrated in Figure 5 for the year 2020. As 2020 is not a simulation year, these results are independent of the scenario considered; comprehensive results for subsequent years and all analyzed scenarios are provided in the Supporting Information. The footprint values for the conventional (BF-BOF, EAF) and DRI-EAF-NG routes fall within the reference ranges,^30^ while there is a limited availability of literature for comparing results for emergent technologies, due to their current limited adoption. The total GHG footprint is decomposed by economic sector and by scope (i.e., Scope 1 for direct emissions of the technology, Scope 2 for emissions embedded in electricity consumption, and Scope 3 for upstream emissions) to identify at which stage of the supply chain the emissions embodied in a tonne of steel are generated.

**Figure 5:**
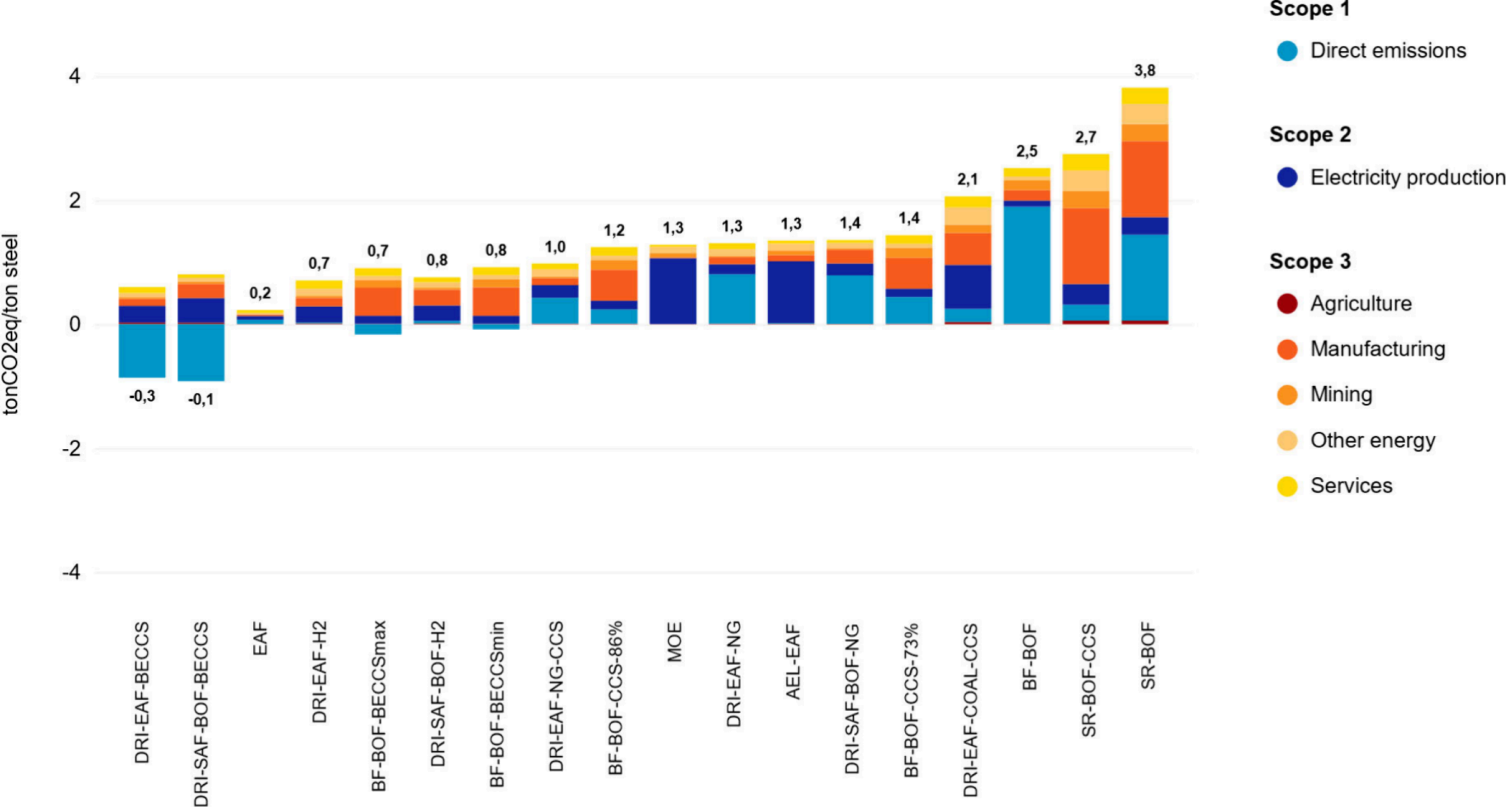
GHG footprint of steel production technologies in the EU for the year 2020 with contributions by technology and scope.

For BF-BOF technologies, the conventional BF-BOF route yields a carbon footprint of 2.52 ton_CO_2_eq_/ton_steel_, primarily attributable to direct emissions from the coal-based blast furnace process. When carbon capture and storage (CCS) is applied, the reduction in direct emissions more than offsets the increase in scope 2 and scope 3 emissions associated with the additional electricity demand of the CCS system. As a result, carbon intensity falls to 1.44 and 1.24 ton_CO2eq_/ton_steel_ for the BF-BOF-CCS-73% and BF-BOF-CCS-86% routes, respectively. The integration of bioenergy with CCS (BECCS) reduces the footprint further, to 0.83 (BF-BOF-BECCSmin) and 0.74 (BF-BOF-BECCSmax) ton_CO2eq_/ton_steel_, underscoring the mitigation potential of combining fuel substitution with negative emission technology.

The influence of the fuel choice is equally apparent in the DRI-EAF routes, where switching from natural gas (1.31 ton_CO_2_eq_/ton_steel_) to green hydrogen (0.70 ton_CO_2_eq_/ton_steel_) substantially reduces GHG intensity. As with the conventional route, integrating CCS into the natural gas-based DRI process yields a reduction in emissions to 0.98 ton_CO_2_eq_/ton_steel_. DRI-based routes employing bioenergy as fuel in combination with CCS achieve net negative carbon footprints of −0.12 and −0.26 ton_CO_2_eq_/ton_steel_ for DRI-SAF-BOF-BECCS and DRI-EAF-BECCS, respectively, as captured emissions exceed those generated. Among innovative electrolysis-based steelmaking routes, MOE and AEL-EAF achieve comparable emission intensities of 1.28 and 1.35 ton_CO_2_eq_/ton_steel_, respectively.

For electricity-intensive technologies, notably DRI-EAF- and electrolysis-based routes, Scope 2 emissions constitute a significant share of the total footprint, meaning that electricity sector decarbonization will have a beneficial effect on their carbon intensity. When the EU electricity mixes provided by GCAM are applied, the high penetration of renewables achieved by 2050 (Figure 6a) enables a reduction of approximately 40% in the carbon intensity of DRI-EAF-H2 and of more than 80% for MOE and AEL-EAF.

**Figure 6:**
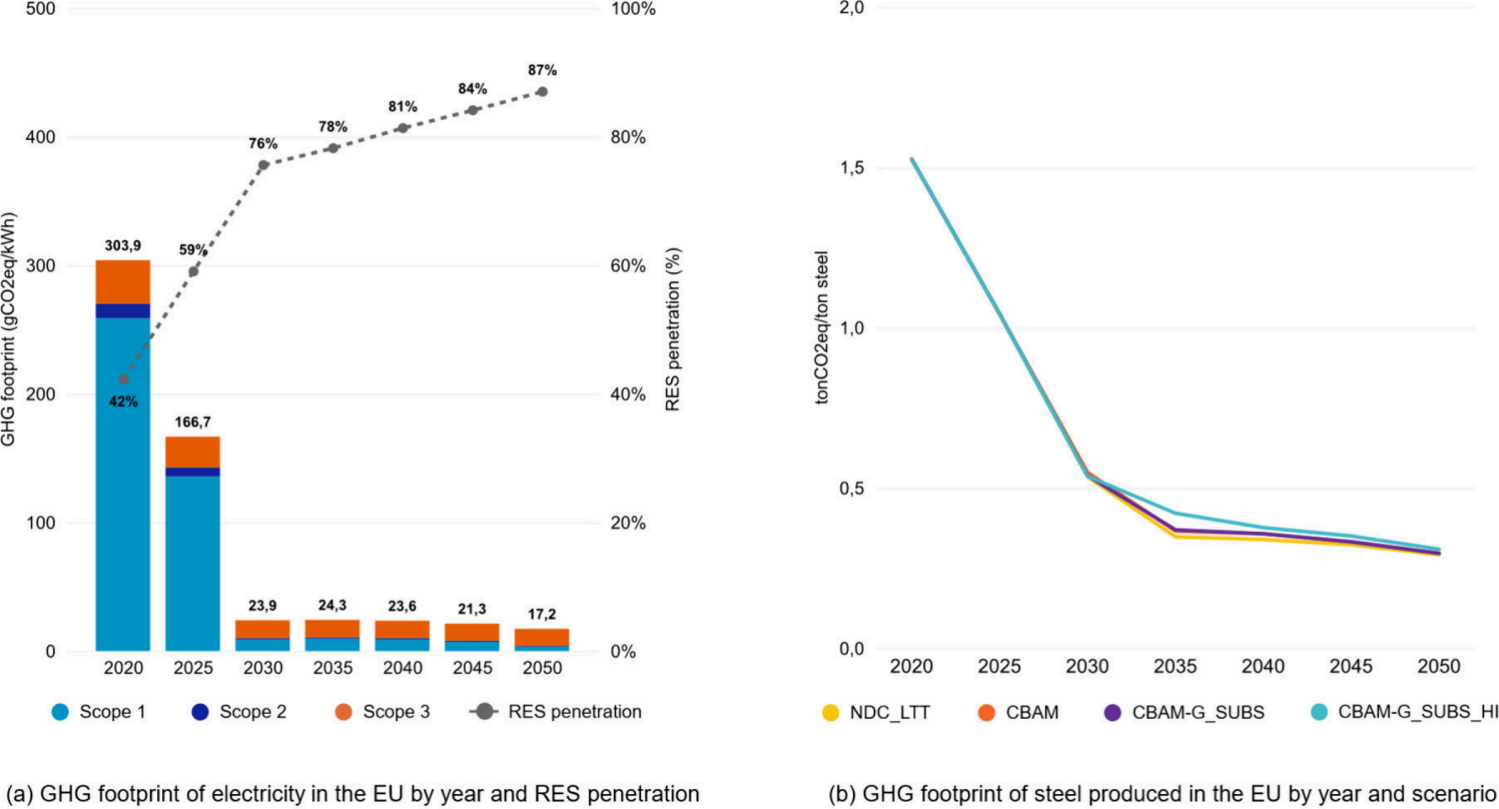
(a, b) GHG footprint of electricity and steel in the EU by year.

#### 3.2.2 Environmental Impact of Analyzed Scenarios

Integrating GCAM results into MARIO allows the GHG footprint of the average tonne of EU-produced steel to be traced as both the steel and electricity sectors decarbonize. As shown in Figure 6b, the average carbon intensity of EU steel production declines substantially across all scenarios, falling from 1.53 in 2020 to 0.30 ton_CO_2_eq_/ton_steel_ in 2050. This outcome is directly attributable to the widespread adoption of low-carbon steelmaking technologies, the growing penetration of renewables in EU electricity generation, and the progressive phase-out of ‘high’ and ‘medium’ emissions production routes.

While the 2050 carbon intensity is practically identical across all scenarios, the trajectories leading to this outcome differ depending on the scenario considered. Counterintuitively, CBAM scenarios do not yield a more rapid decline in the carbon footprint of EU-produced steel relative to that of the NDC_LTT scenario. The largest divergence is observed in 2035, where the CBAM-G_SUBS_HI and NDC_LTT scenarios record GHG intensities of 0.42 and 0.35 ton_CO_2_eq_/ton_steel_, respectively. Although the former scenario achieves higher absolute volumes of low-emission steel production, the share of low-emissions steel in the total production mix (i.e., the ratio between low-emissions and total steel production) is slightly lower, owing to the increased overall production volumes. Consequently, total domestic embedded emissions (Mton_CO2eq_), obtained by multiplying the average GHG footprint of EU steel by total domestic consumption, are also higher in the CBAM-G_SUBS_HI scenario than in the NDC_LTT scenario in that year, as illustrated in Figure 7.

**Figure 7:**
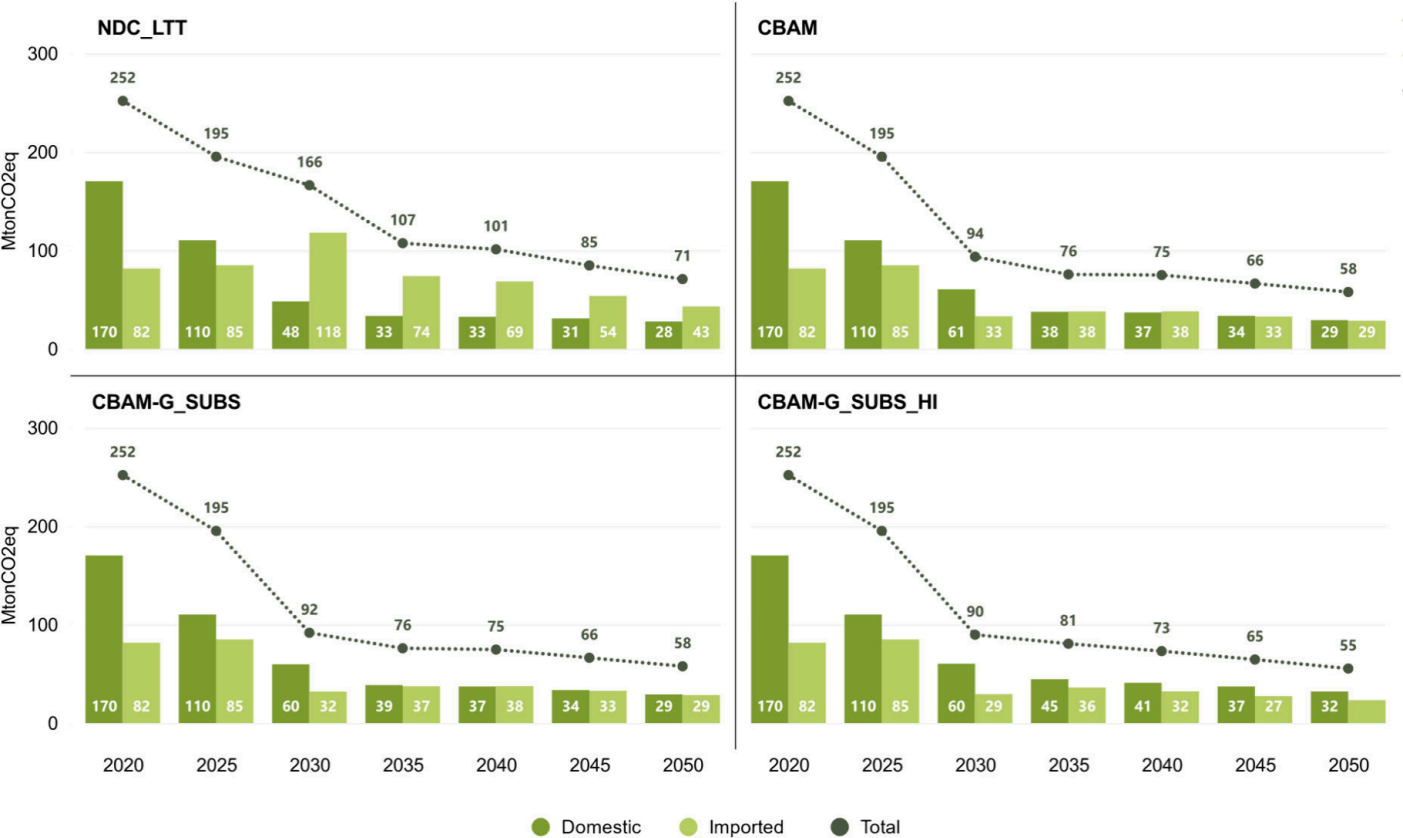
Domestic and imported GHG emissions embedded in steel consumption in the EU by scenario.

The effect of CBAM becomes apparent when considering the GHG footprint of steel consumed in the EU, which comprises both domestically produced and imported steel. Figure 8 decomposes this footprint by scope for both origin categories, revealing that all CBAM scenarios achieve a footprint approximately half that of the NDC_LTT scenario in 2030 (0.62–0.64 versus 1.13 ton_CO_2_eq_/ton_steel_). This result stems from two concurrent effects: a lower emissions intensity of imported steel under CBAM and a contraction in import volumes that shifts the consumption mix toward less carbon-intensive domestic steel. As shown in Figure 7, CBAM, whether or not supplemented by green steel subsidies, is effective at curbing emissions associated with steel imported into the EU. As a result, maintaining broadly equivalent total consumption levels, the CBAM scenarios achieve reductions in total GHG emissions embedded in steel consumption of up to −46% in 2030 and up to −23% in 2050 relative to the NDC_LTT scenario. Moreover, over the full thirty-year modeling horizon, cumulative embedded emissions under CBAM scenarios are approximately 25% lower than under the baseline NDC_LTT scenario.

**Figure 8:**
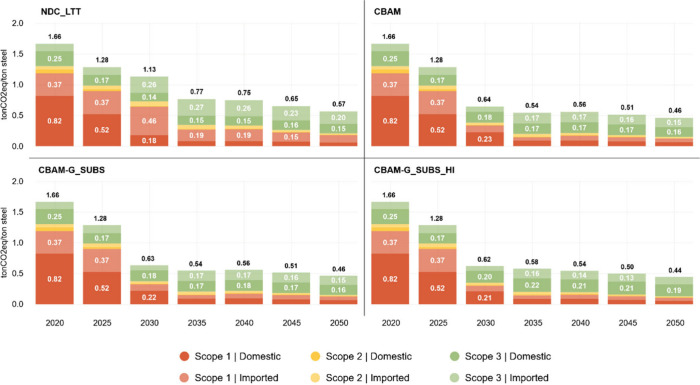
GHG footprint of EU-consumed steel by scenario and year with contributions by scope of imported and domestic steel.

Finally, within the CBAM scenarios, total embedded emissions remain broadly similar, indicating that, while the subsidies modeled in the CBAM-G_SUBS and CBAM-G_SUBS_HI scenarios enhance the competitiveness of the European steel industry (as discussed in section 3.1), they do not produce a meaningful reduction in greenhouse gas emissions.

### 3.3 Discussion and Implications

The decarbonization of the European steel industry, while essential for reducing sectoral GHG emissions, risks undermining the competitiveness of EU steel on both domestic and international markets and driving carbon leakage. To address these risks, the EU introduced CBAM to impose a levy on carbon-intensive steel imports. Given that the EU is the world’s largest steel importer, CBAM is likely to exert significant effects on exporting countries. For this reason, in recent years a growing body of literature has sought to assess the economic and environmental impacts of CBAM on the global steel sector and on specific national industries. The present study contributes to this literature by analyzing the consequences of CBAM and complementary green steel subsidies for the EU, coupling the GCAM and MARIO/Exiobase modeling frameworks to examine policy-driven changes in production, consumption, and trade volumes, as well as their implications for the GHG footprint of steel produced and consumed in the region.

The GCAM results indicate that CBAM exerts limited influence on long-term production, consumption, and trade levels, primarily due to carbon pricing being adopted in regions outside the EU. CBAM’s relevance is instead concentrated in the short term, particularly around 2030, where it appears capable of partially offsetting the loss of competitiveness that the European steel industry would otherwise face, thereby limiting the decline in production and the rise in imports. In the long term, the only policy instrument among those analyzed that yields sustained effects is the operational cost subsidy for green steel technologies modeled in the CBAM-G_SUBS_HI scenario, which drives a more pronounced increase in steel production, exceeding 2020 levels, and exports. These findings imply that capital subsidies, as applied in the CBAM-G_SUBS scenario, while important as an initial investment incentive, contribute relatively little to reducing the levelized cost of steel production over the medium to long-term, and therefore do not improve EU competitiveness beyond what CBAM alone achieves. Meaningful and sustained gains in production and exports only occur when operational costs are addressed, as occurs in the CBAM-G_SUBS_HI scenario, suggesting that improving long-term competitiveness requires ongoing operational support rather than one-off capital injections.

The impact of CBAM is also evident in the type of steel (low, medium, high emissions) that is produced, consumed, and traded within the EU. Between 2025 and 2035, CBAM scenarios are projected to increase EU low-emission steel production relative to the baseline (NDC_LTT) scenario, while the level of high- and medium-emission steel production is predicted to remain unchanged. This indicates that the increase in production volumes observed in CBAM scenarios around 2030 is associated with the adoption of low-carbon, primarily scrap-based steel production technologies within the EU. The higher production levels occurring in the CBAM scenarios in the short term imply that, despite the production of more low-emission steel, the carbon footprint of steel produced in the EU is higher than that in the NDC_LTT scenario. Nevertheless, total emissions incorporated into steel consumption, which is almost identical for all scenarios, begin to decline around 2030 in all CBAM scenarios, implying lower cumulative emissions by 2050 than those in the baseline scenario. This is due to the reduction in emissions associated with imported steel as CBAM scenarios simultaneously reduce import volumes and increase the share of low-emission steel in imports.

The beneficial effect of CBAM on domestic production could, however, be undermined if exporting countries selectively redirect low-emission steel toward the EU market while continuing to supply conventional steel to other markets. The modeling framework employed in this study cannot capture such behavior, as it does not simulate bilateral trade flows but instead assumes that all exporting countries contribute to a global trade pool in proportion to their domestic production mix. Future work should therefore incorporate bilateral trade flows to refine the scenario outcomes. Further extensions could include sensitivity analyses on key parameters, such as scrap availability and the global average carbon price, which may influence the estimated effects of CBAM.

## Supplementary Material



## Data Availability

The study is accompanied by Supporting Information containing some inputs data and the full set of results from the study. The EXIOBASE database extended with steelmaking technologies adopted in the MARIO model is available on Zenodo at the following link: 10.5281/zenodo.10843374. The GCAM version applied in this study is openly available through GitHub: https://github.com/russellhz/gcam-core/tree/Study9_IAMCOMPACT.

## Notes

Table of concordance between regions in GCAM and MARIO/Exiobase input data for steelmaking technologies with references; Data underlying Figure 2 in the Manuscript; Data underlying Figures 3 and 4 in the Manuscript; Data underlying Figure 5 in the Manuscript; Data underlying Figure 6 in the Manuscript; Data underlying Figure 7 in the Manuscript; Full set of results obtained from GCAM (XLSX)

## Abbreviations

BF-BOF: Blast Furnace-Basic Oxygen Furnace
CBAM: Carbon Border Adjustment Mechanism
CGE: Computable General Equilibrium
DR: Direct Reduction
EAF: Electric Arc Furnace
ETS: Emission Trading System
EU: European Union
GCAM: Global Change Analysis Model
GDP: Gross Domestic Product
GHG: Greenhouse Gas
GTAP: Global Trade Analysis Project
ICIO: Inter-Country Input-Output tables
MARIO: Multifunctional Assessment of Regions through Input-Output
OECD: Organisation for Economic Co-operation and Development
